# A group of *Populus trichocarpa* DUF231 proteins exhibit differential *O*-acetyltransferase activities toward xylan

**DOI:** 10.1371/journal.pone.0194532

**Published:** 2018-04-04

**Authors:** Ruiqin Zhong, Dongtao Cui, Zheng-Hua Ye

**Affiliations:** 1 Department of Plant Biology, University of Georgia, Athens, GA, United States of America; 2 Department of Chemistry, University of Georgia, Athens, GA, United States of America; Iowa State University, UNITED STATES

## Abstract

Wood represents the most abundant biomass produced by plants and one of its major components is acetyl xylan. Acetylation in xylan can occur at *O*-2 or *O*-3 of a xylosyl residue, at both *O*-2 and *O*-3 of a xylosyl residue, and at *O*-3 of a xylosyl residue substituted at *O*-2 with glucuronic acid. Acetyltransferases responsible for the regiospecific acetylation of xylan in tree species have not yet been characterized. Here we report the biochemical characterization of twelve *Populus trichocarpa* DUF231-containing proteins, named PtrXOATs, for their roles in the regiospecific acetylation of xylan. The PtrXOAT genes were found to be differentially expressed in *Populus* organs and among them, *PtrXOAT1*, *PtrXOAT2*, *PtrXOAT9* and *PtrXOAT10* exhibited the highest level of expression in stems undergoing wood formation. Activity assays of recombinant proteins demonstrated that all twelve PtrXOAT proteins were able to transfer acetyl groups from acetyl CoA onto a xylohexaose acceptor with PtrXOAT1, PtrXOAT2, PtrXOAT3, PtrXOAT11 and PtrXOAT12 having the highest activity. Structural analysis of the PtrXOAT-catalyzed reaction products using ^1^H NMR spectroscopy revealed that PtrXOAT1, PtrXAOT2 and PtrXOAT3 mediated 2-*O*- and 3-*O*-monoacetylation and 2,3-di-*O*-acetylation of xylosyl residues and PtrXOAT11 and PtrXOAT12 only catalyzed 2-*O*- and 3-*O*-monoacetylation of xylosyl residues. Of the twelve PtrXOATs, only PtrXOAT9 and PtrXOAT10 were capable of transferring acetyl groups onto the *O*-3 position of 2-*O*-glucuronic acid-substituted xylosyl residues. Furthermore, when expressed in the Arabidopsis *eskimo1* mutant, PtrXOAT1, PtrXAOT2 and PtrXOAT3 were able to rescue the defects in xylan acetylation. Together, these results demonstrate that the twelve PtrXOATs are acetyltransferases with different roles in xylan acetylation in *P*. *trichocarpa*.

## Introduction

Plants convert solar energy into chemical energy (fixed carbon) through photosynthesis, and the bulk of this fixed carbon is stored in the form of wood and fibers. Of the estimated fixation of 56 billion metric tons of carbon by land plants every year, about half is stored in tree species [1]. Therefore, wood is the most abundant reservoir of fixed carbon and stored energy in plants. Deciphering the biochemical mechanisms underlying the biosynthesis of wood components will provide fundamental insights into how plants convert fixed carbon from photosynthesis into long-term stored energy in the form of wood and fibers. Considering the many applications of wood as raw materials for industrial uses and potentially for bioenergy production, understanding how wood is synthesized may also offer novel means for modification of wood components better suited to our needs.

Wood is mainly composed of cellulose, hemicelluloses (xylan and glucomannan) and lignin, the biosynthesis of which involves coordinated regulation of many biosynthetic pathway genes during wood formation. Previous transcriptomic analyses in tree species have identified a number of wood-associated genes involved in transcriptional regulation of wood formation and secondary wall biosynthesis [2–5]. Further genetic and biochemical analyses in the model woody species *Populus* have demonstrated that cellulose biosynthesis during wood formation is mediated by wood-associated cellulose synthases [2,6,7], glucomannan biosynthesis is catalyzed by cellulose synthase-like A glycosyltransferases [6,8], and lignin biosynthesis is carried out through the phenylpropanoid pathway involving at least 10 enzymes and the monolignol polymerizing enzymes including laccases [9,10]. In contrast, much less is known about the genes participating in xylan biosynthesis in woody species.

Previous chemical analyses of xylan structure in tree species have revealed that xylans in the wood of both gymnosperms and angiosperms are composed of a linear chain of β-1,4-linked xylosyl (Xyl) residues that are often substituted at *O*-2 with 4-*O*-methylglucuronic acid (MeGlcA) side chains [11]. The reducing end sequence of xylan differs from the backbone by having a unique tetrasaccharide sequence consisting of β-D-Xyl*p*-(1→3)-α-L-Rha*p*-(1→2)-α-D-Gal*p*A-(1→4)-D-Xyl*p* [12–15]. In addition, xylan in angiosperm wood is heavily acetylated at *O*-2 and *O*-3 and xylan in gymnosperm wood is substituted with arabinosyl residues in addition to MeGlcA [11]. In *Arabidopsis thaliana*, a number of genes participating in xylan biosynthesis have been identified and characterized; these include members of GT47 (IRX10 and IRX10L) and GT43 (IRX9 and IRX14) families for xylan backbone elongation, members of GT8 (IRX8 and PARVUS) and GT47 (FRA8) families for biosynthesis of xylan reducing end sequence, members of GT8 (GUXs) family for addition of GlcA side chains, members of DUF579 (GXMs) family for methylation of GlcA side chains, and members of RWAs and DUF231-containing proteins (ESK1 and its close homologs) for xylan acetylation [16]. A number of close homologs of Arabidopsis xylan biosynthetic genes have been identified in the genome of *Populus trichocarpa* and were shown to be preferentially expressed during wood formation [2–5], implicating their roles in xylan biosynthesis. Among them, it has been demonstrated that five PtrGT43 members are functional orthologs of IRX9 or IRX14 essential for xylan backbone elongation [17,18], PoGT47C, PoGT8D and PoGT8E/PoGT8F are functional orthologs of FRA8, IRX8 and PARVUS, respectively, required for the biosynthesis of xylan reducing end sequence [15,17,19], four DUF579 members (PtrGXMs) are methyltransferases catalyzing the methylation of GlcA side chains [20], and four RWA members are putative transporters essential for xylan acetylation [21]. Acetyltransferases responsible for xylan acetylation during wood formation in tree species have not yet been characterized.

Xylan from angiosperms has been shown to be acetylated at various positions of Xyl residues, including 2-*O*- and 3-*O*-monoacetylation and 2,3-di-*O* acetylation of Xyl residues as well as 3-*O*-acetylation of 2-*O*-GlcA-substituted Xyl residues. The degree of acetyl substitution in xylans (the relative molecular ratio of total acetyl groups versus total Xyl residues) from aspen, birch, beech, Eucalyptus and *Paulownia* ranges from 0.39 to 0.61 [22–25]. Xylan is believed to interact with cellulose to form a reinforced structural network essential for wood strength and xylan acetylation plays an important role in the formation of this network. The degree of xylan acetylation has been shown to strongly affect the solubility properties and the water content of xylan and the adsorption of xylan to cellulose [26,27]. In addition, it has been demonstrated that a reduction in the degree of xylan acetylation impedes the deposition of cellulose and xylan and alters the assembly of secondary walls leading to retarded plant growth [28]. On the other hand, the presence of acetyl groups in xylan contributes to the recalcitrance of biomass during its conversion into biofuels, i.e., the acetyl groups in xylan obstruct the enzymatic digestion of biomass into fermentable sugars and the acetic acid generated from pretreatment of biomass inhibits the growth of yeast used for fermentation of sugars [29,30]. Therefore, identification and characterization of acetyltransferases responsible for xylan acetylation in tree species may have practical implications in genetic improvement of wood biomass for biofuel production.

In Arabidopsis, nine members belonging to the ESK1 group of the DUF231-containing protein family have been demonstrated to be acetyltransferases catalyzing the regiospecific acetylation of xylan [28,31–35]. In this report, we identified sixty-four DUF231 genes in the genome of *P*. *trichocarpa*, of which twelve are phylogenetically grouped together with the Arabidopsis xylan acetyltransferases. We studied the expression patterns of these twelve DUF231 genes and investigated the biochemical properties of their recombinant proteins. We found that four of them, *PtrXOAT1*, *PtrXOAT2*, *PtrXOAT9* and *PtrXOAT10*, were highly expressed in stems undergoing secondary growth. We demonstrated that among the twelve recombinant PtrXOAT proteins, PtrXOAT1, PtrXOAT2 and PtrXOAT3 exhibited the highest acetyltransferase activity catalyzing 2-*O*- and 3-*O*-monacetylation and 2,3-di-*O* acetylation of Xyl residues, and PtrXOAT9 and PtrXOAT10 were the only acetyltransferases mediating 3-*O*-acetylation of 2-*O*-GlcA-substituted Xyl residues. Our results indicate that the twelve PtrXOATs are acetyltransferases with differential activities involved in xylan acetylation in *Populus*.

## Results

### Xylan from *P*. *trichocarpa* stems is predominantly 2-*O*- and 3-*O*-monoacetylated and 2,3-di-*O*-acetylated

**Fig 1:**
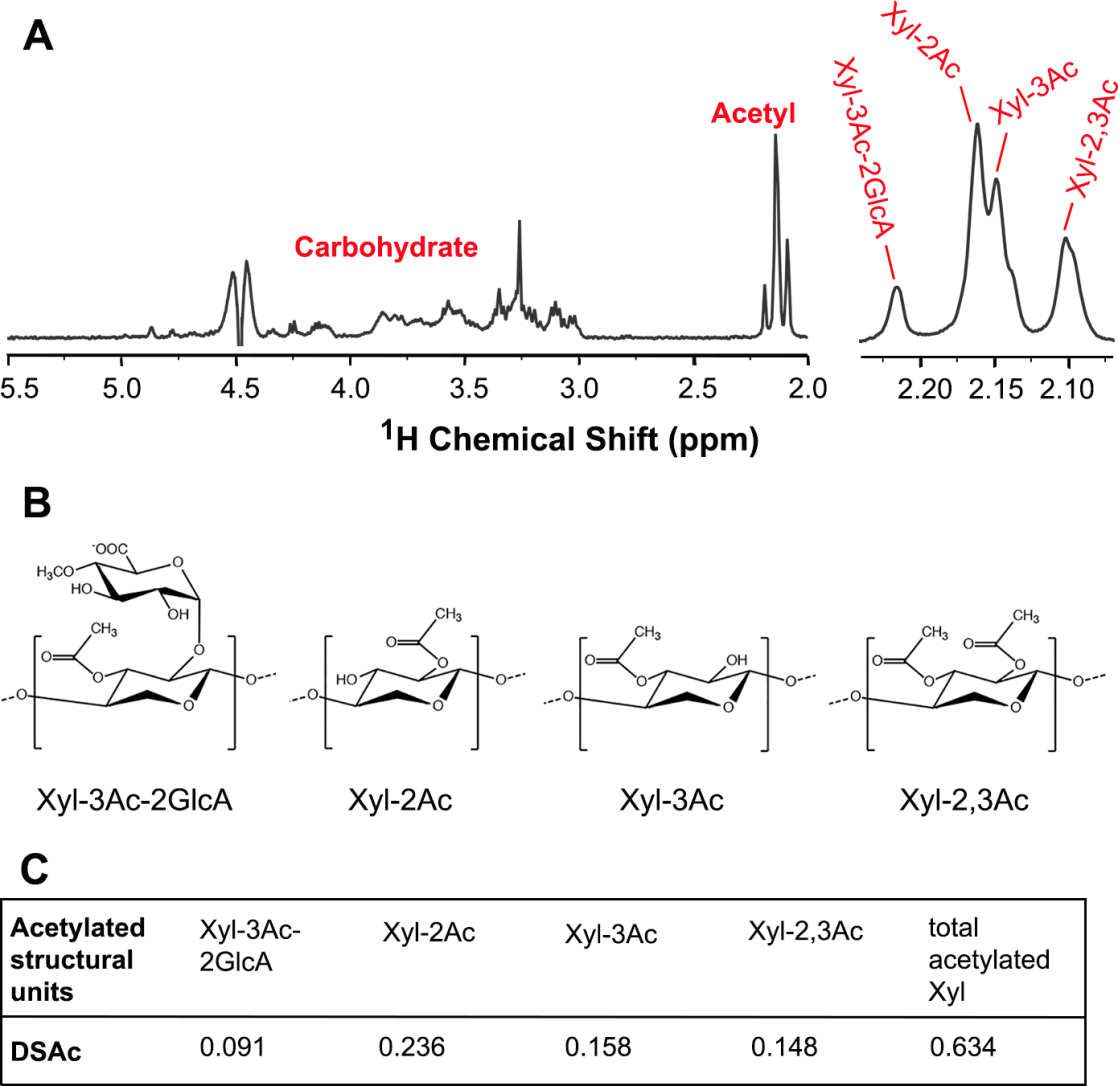
Structural analysis of acetyl substitutions in *Populus* xylan. Xylan was isolated from *Populus* stems and determined for the presence of acetyl groups at different positions of Xyl residues using ^1^H NMR spectroscopy. (A) ^1^H NMR spectrum of *Populus* xylan showing the resonance signals corresponding to carbohydrate and acetyl groups (left panel) and the enlarged region of acetyl signal peaks attributed to acetylated Xyl residues (right panel). (B) Diagrams of four different types of acetyl substitutions of a xylosyl residue, including Xyl-3Ac-2GlcA (2-*O*-GlcA-substituted Xyl residue acetylated at *O*-3), Xyl-2Ac (Xyl residue monoacetylated at *O*-2), Xyl-3Ac (Xyl residue monoacetylated at *O*-3), and Xyl-2,3Ac (Xyl residue diacetylated at *O*-2 and *O*-3). (C) Integration analysis of the degree of acetyl substitution in *Populus* xylan. The degree of acetyl substitution in xylan was determined by integration of resonance signals corresponding to each type of acetylated Xyl residues relative to the signals for carbohydrate in the ^1^H NMR spectra of *Populus* xylan (B). The data were the average of three independent experiments.

To study acetyltransferases involved in xylan acetylation in *P*. *trichocarpa*, we first determined the acetylation pattern of xylan from *P*. *trichocarpa* stems. DMSO-extracted xylan was digested with endo-β-xylanase and the released xylooligomers were analyzed for their acetylation pattern using ^1^H nuclear magnetic resonance (NMR) spectroscopy (Fig 1). The ^1^H NMR spectra exhibited resonances between 3.0 to 5.5 ppm corresponding to Xyl residues and resonances between 2.0 and 2.25 ppm that are characteristic of acetyl groups (Fig 1A, left panel). Further examination of the resonances for acetyl groups showed four distinct signal peaks at 2.10 ppm, 2.15 ppm, 2.17 ppm and 2.22 ppm (Fig 1A, right panel). The chemical shifts of these signal peaks are identical to those of acetyl xylan from *Eucalyptus globulus*, which were previously assigned to 2,3-di-*O*-acetylated (Xyl-2,3Ac; 2.10 ppm), 3-*O*-monoacetylated (Xyl-3Ac; 2.15 ppm), 2-*O*-monoacetylated (Xyl-2Ac; 2.17 ppm) and 3-*O*-acetylated 2-*O*-GlcA-substituted (Xyl-3Ac-2GlcA; 2.22 ppm) Xyl residues [36] (Fig 1B). The existence of these four resonance peaks in the NMR spectra of *P*. *trichocarpa* xylan indicates the presence of these four acetylated structural units. Since the resonances for the acetylated structural units were well separated from those for the xylosyl units, they were used for measurement of the degree of xylan acetylation. Integration analysis of the NMR resonance signals demonstrated that the degree of acetyl substitution in xylan from *P*. *trichocarpa* stems was 63%, including 23.6% attributed to Xyl-2Ac, 15.8% to Xyl-3Ac, 14.8% to Xyl-2,3Ac and 9.1% to Xyl-3Ac-2GlcA (Fig 1C). The structural analysis indicates that xylan from *P*. *trichocarpa* stems is heavily acetylated with the predominant acetylated structural units being Xyl-2Ac, Xyl-3Ac and Xyl-2,3Ac.

### Identification and expression analysis of twelve *P*. *trichocarpa* DUF231 genes that are close homologs of Arabidopsis XOATs

**Fig 2:**
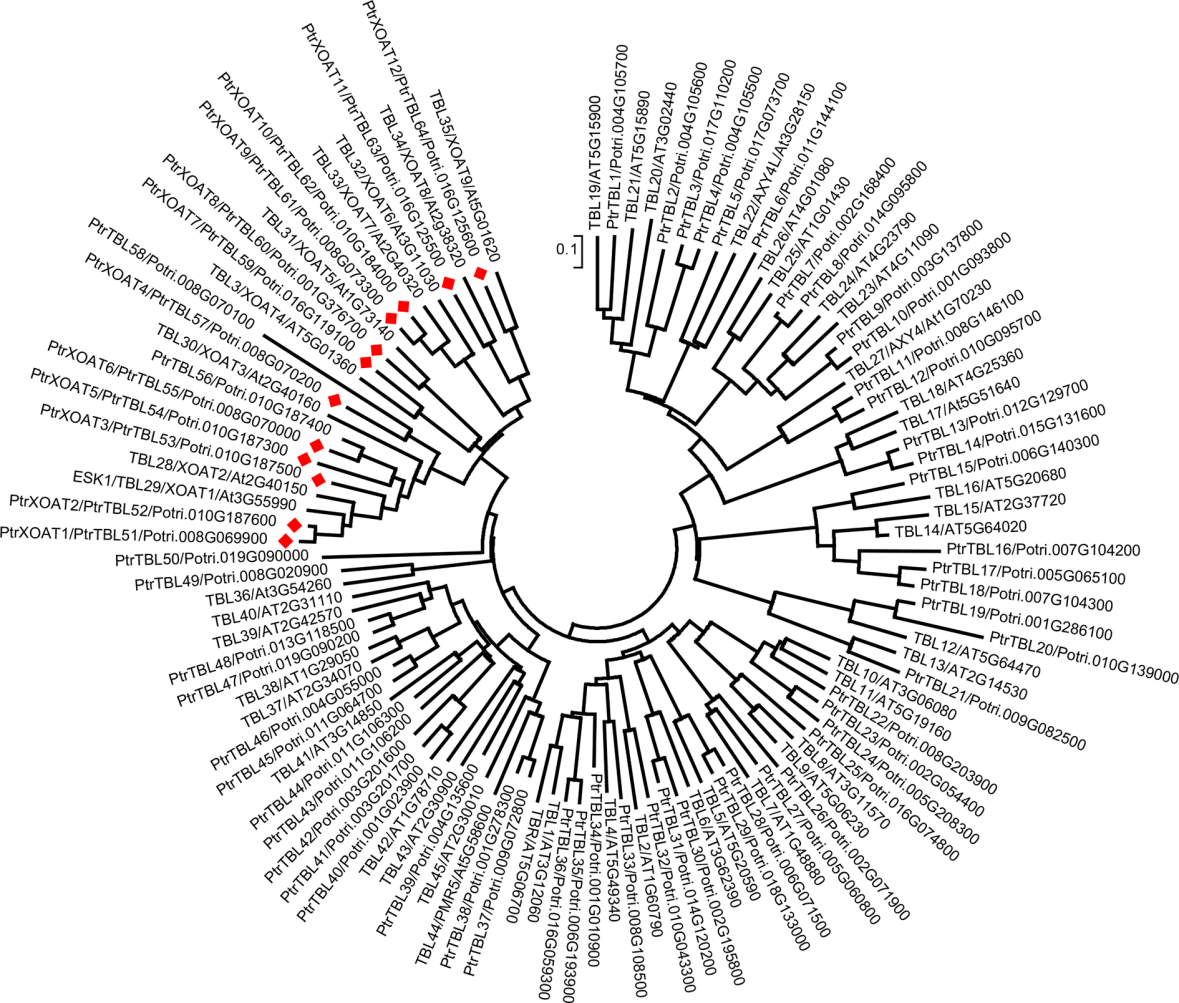
Phylogenetic analysis of DUF231 genes in *P*. *trichocarpa*. The protein sequences of Arabidopsis DUF231 genes were used to blast search for DUF231 homologs in the genome of *P*. *trichocarpa*. Protein sequences from both *P*. *trichocarpa* and Arabidopsis were used to construct the phylogenetic tree with the neighbor-joining algorithm. Each DUF231 gene was labeled with its TBL name together with its gene locus identifier. The 0.1 scale denotes 10% change. The 12 *Populus* DUF231 genes studied in this report were named PtrXOATs and marked with red diamonds.

**Fig 3:**
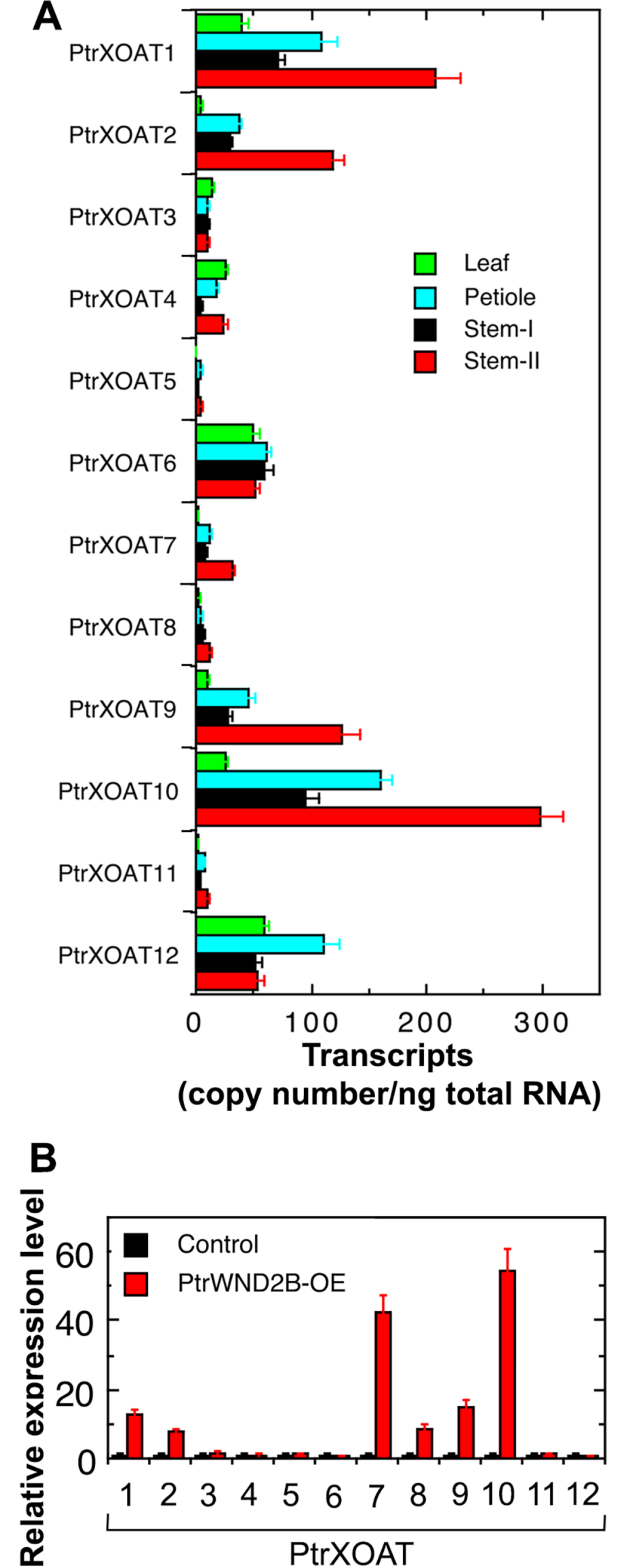
Expression analysis of PtrXOAT genes. (A) Quantitative PCR analysis of PtrXOAT gene expression in leaves, petioles, and stems with primary growth (Stem-I) and stems with secondary growth (Stem-II). The data were the average of three biological replicates. (B) Induction of expression of PtrXOAT genes by the wood-associated secondary wall NAC master switch PtrWND2B. Total RNAs from the control (transformed with an empty vector) and PtrWND2B overexpressors (PtrWND2B-OE) were examined for expression of PtrXOAT genes by quantitative PCR analysis. The expression level in the control was set to 1. The data were the average of three biological replicates.

Previous studies in Arabidopsis have demonstrated that nine DUF231 genes, including *ESK1* and its close homologs (*TBL3*, *TBL28*, *TBL30*, *TBL31*, *TBL32*, *TBL33*, TBL34 and *TBL35*), encode xylan acetyltransferases, XOATs [28,31–35]. Sequence analysis identified a total of sixty-four DUF231 genes in the genome of *P*. *trichocarpa*, and among them, twelve were grouped together with Arabidopsis XOATs (Fig 2) and thus named PtrXOATs. It should be noted that based on their deduced partial amino acid sequences, the Potri.010G187400 (*TBL56*) and Potri.008G070100 (*TBL58*) genes were grouped together with PtrXOATs. However, sequencing of their transcripts revealed premature stop codons in the coding region, and hence they are pseudogenes and were not included in this study. To find out which PtrXOAT genes were preferentially expressed in stems in which xylan-containing wood is formed, we performed quantitative reverse transcription-polymerase chain reaction (RT-PCR) analysis of PtrXOAT expression in leaves, petioles and stems (Fig 3A). Stem tissues at two different developmental stages characterized by primary growth (stem-I) and secondary growth (stem-II) were analyzed. Although the expression of all twelve PtrXOAT genes was detected in all four tissues examined, four of them, *PtrXOAT1*, *PtrXOAT2*, *PtrXOAT9* and *PtrXOAT10* had the highest accumulation of transcripts in stems undergoing secondary growth.

To determine which PtrXOATs might be involved in secondary wall biosynthesis during wood formation, we investigated their expression levels in the transgenic *Populus* lines overexpressing PtrWND2B, a wood-associated NAC domain transcriptional master switch activating secondary wall biosynthesis in *Populus* wood [37]. Quantitative PCR analysis showed that the expression of six of them, including *PtrXOAT1*, *PtrXOAT2*, *PtrXOAT7*, *PtrXOAT8*, *PtrXOAT9* and *PtrXOAT10*, was induced by PtrWDN2B overexpression (Fig 3B), indicating that they are PtrWND2B-regulated downstream genes involved in secondary wall biosynthesis. Consistent with their putative roles in xylan biosynthesis, all twelve PtrXOAT proteins were predicted to be Golgi-localized type II transmembrane proteins with one transmembrane helix (S1 Fig) by the TMHMM2.0 program (http://www.cbs.dtu.dk/services/TMHMM/) and the Golgi Predictor program (http://ccb.imb.uq.edu.au/golgi/).

### PtrXOATs exhibit differential *O*-acetyltransferase activities toward xylan

**Fig 4:**
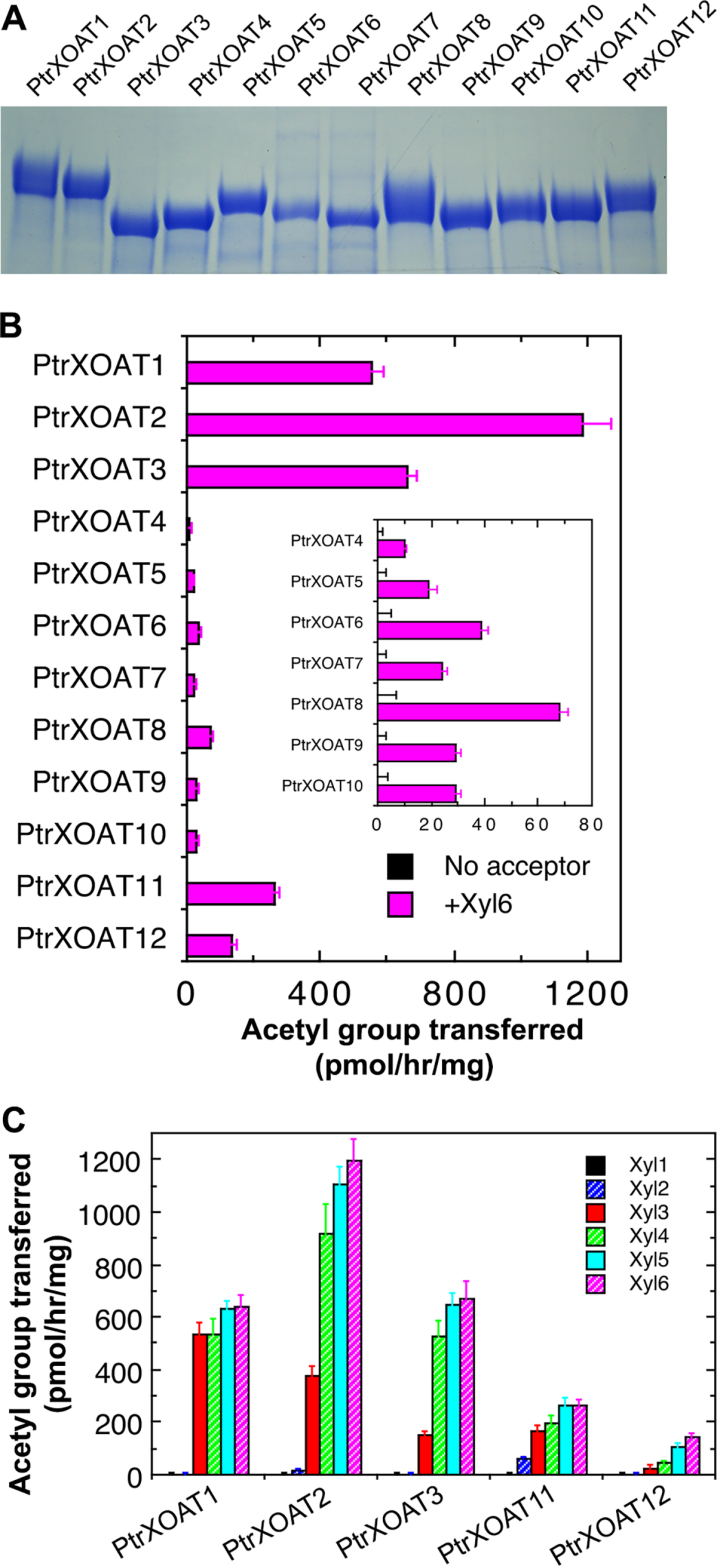
Production and xylan acetyltransferase activity assay of recombinant PtrXOAT proteins. His-tagged recombinant PtrXOAT proteins without the N-terminal transmembrane sequence were expressed in the secreted form in mammalian HEK293F cells and purified for activity assay. (A) SDS-polyacrylamide gel electrophoresis showing the purified recombinant PtrXOAT proteins. Proteins were detected by staining with Coomassie Blue. (B) Assay of recombinant PtrXOAT proteins for xylan acetyltransferase activities. PtrXOATs were incubated with ^14^C-labeled acetyl CoA and the Xyl_6_ acceptor for examination of their ability to transfer the radiolabeled acetyl group from acetyl CoA onto the Xyl_6_ acceptor. PtrXOATs incubated with ^14^C-acetyl CoA without Xyl_6_ were used as controls (No acceptor). (C) Acetyltransferase activities of PtrXOATs toward xylooligomers with different degree of polymerization (DP). The data were the average of three biological replicates.

To investigate their enzymatic activities and biochemical properties, His-tagged recombinant PtrXOAT proteins without the N-terminal transmembrane domain were expressed in the secreted form in human embryonic kidney (HEK) 293F cells. The recombinant proteins were purified by passing the culture medium through a nickel resin column and detected by gel electrophoresis and Coomassie Blue staining (Fig 4A). The purified recombinant PtrXOAT proteins were incubated with ^14^C-labeled acetyl-CoA and the xylohexaose acceptor for detection of xylan acetyltransferase activities. It was revealed that all twelve recombinant PtrXOATs were able to transfer the ^14^C-labeled acetyl groups from acetyl-CoA onto xylohexaose (Fig 4B). Among them, PtrXOAT1, PtrXOAT2 and PtrXOAT3 exhibited the highest activity, PtrXOAT4 to PtrXOAT10 had a low level of activity, and PtrXOAT11 and PtrXOAT12 showed moderate activity. Mixing PtrXOATs (PtrXOAT4 to PtrXOAT10) having a low activity with PtrXOAT1 that exhibits a high activity in the reaction mixture did not yield a higher activity than the sum of the activity of the two proteins assayed separately (an activity of 400 to 500 pmol/hr/mg for the mixed reactions). Control reactions of PtrXOATs incubated with ^14^C-acetyl-CoA alone without the xylohexaose acceptor did not show any activity (Fig 4B), nor did heat-denatured PtrXOATs incubated with ^14^C-acetyl-CoA and xylohexaose (0 pmol/hr/mg). When mannohexaose or xyloglucan oligosaccharides were used as acceptors in the reactions, the recombinant PtrXOATs were unable to transfer acetyl groups from ^14^C-labeled acetyl-CoA to the oligosaccharides (0 pmol/hr/mg). These results demonstrate that PtrXOATs are *O*-acetyltransferases specifically catalyzing acetyl transfer onto xylan.

We further determined the requirement of the minimal degree of polymerization (DP) of xylooligomers by PtrXOATs having a high acetyltransferase activity, including PtrXOAT1, PtrXOAT2, PtrXOAT3, PtrXOAT11 and PtrXOAT12. It was found that they exhibited no activity toward xylose and xylobiose except that PtrXOAT11 had a low activity toward xylobiose, whereas they were able to transfer acetyl groups onto xylooligomers with a DP of ≥3 (Fig 4C). Although the activities of PtrXOATs increased with the increasing DP of xylooligomers, the level of increase differed among PtrXOATs (Fig 4C). For example, PtrXOAT2 and PtrXOAT3 had a much lower activity toward xylotriose than xylooligomers with a higher DP, whereas PtrXOAT1 displayed a similar level of activity toward xylooligomers with a DP of ≥3 (Fig 4C). These results indicate a variation in the requirement of the minimal DP of xylooligomers by different PtrXOATs for efficient acetylation.

**Fig 5:**
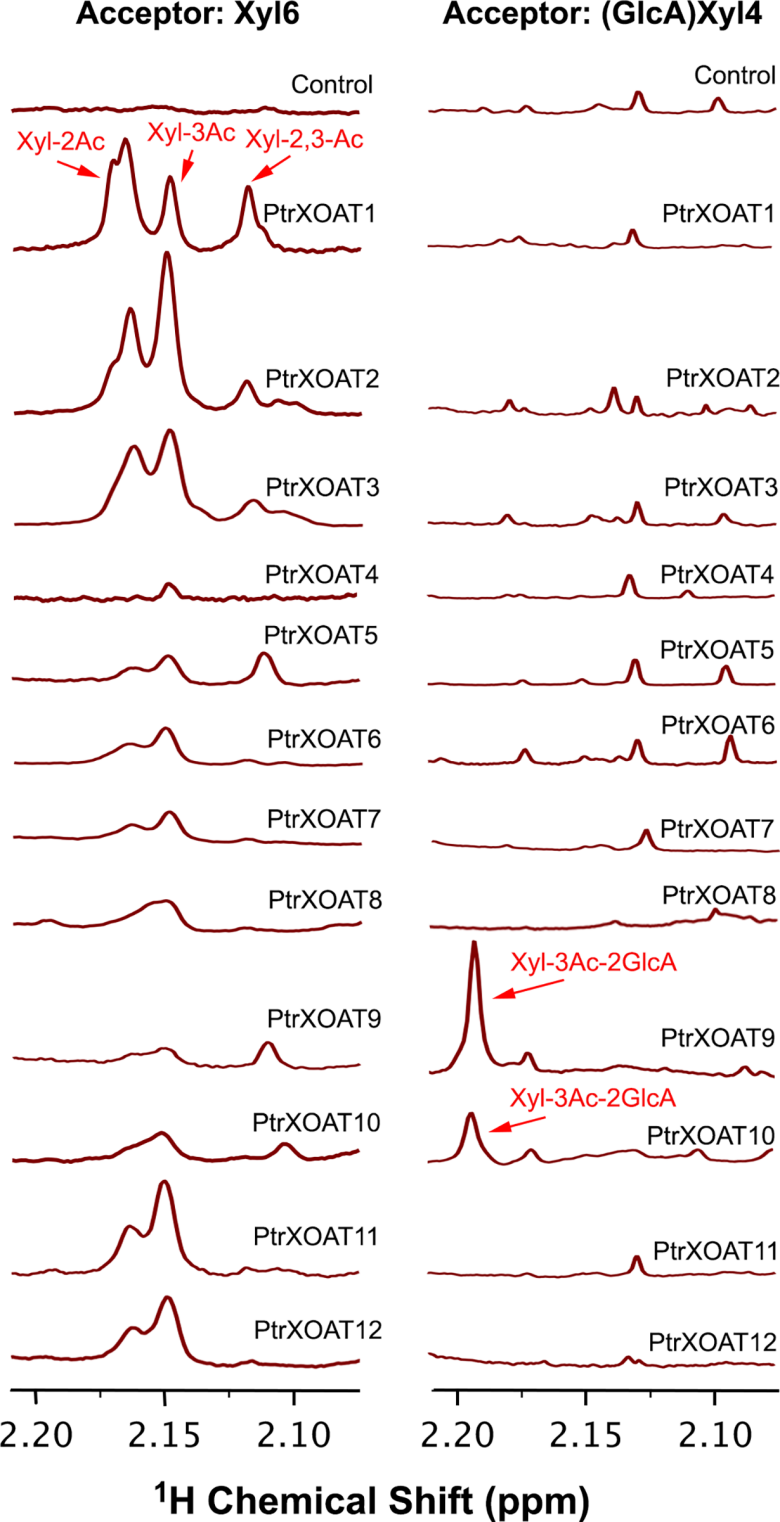
^1^H NMR spectra of PtrXOAT-catalyzed reaction products. Recombinant PtrXOAT proteins were incubated with acetyl CoA and Xyl_6_ (left panel) or (GlcA)Xyl_4_ (right panel), and the reaction products were examined by ^1^H NMR spectroscopy for acetyl substitutions at different positions of Xyl residues. The control reaction contained acetyl CoA and the acceptor only without recombinant proteins. The resonance peaks attributed to Xyl-2Ac, Xyl-3Ac, Xyl-2,3-Ac, and Xyl-3Ac-2GlcA are marked. The additional resonance peaks seen in the spectra of the reactions with (GlcA)Xyl_4_ as the acceptor (right panel) were likely due to the fact that acetyl CoA was not removed from the reaction products. The data shown were representatives of three biological replicates.

The finding that PtrXOATs exhibited differential xylan acetyltransferase activities prompted us to determine whether they also exhibited regiospecific activities for xylan acetylation. To do so, we applied ^1^H NMR spectroscopy to examine the positions of acetyl groups on Xyl residues in the PtrXOAT-catalyzed products. Consistent with the data from the radiolabeled acetyltransferase activity assay as shown in Fig 4B, incubation of PtrXOATs with xylohexaose and acetyl-CoA generated products with resonance signals between 2.1 to 2.2 ppm corresponding to acetyl groups, with PtrXOAT1, PtrXOAT2, PtrXOAT3, PtrXOAT11 and PtrXOAT12 having the highest signals (Fig 5, left panel). There existed three resonance peaks in the reaction products of PtrXOAT1, PtrXOAT2 and PtrXOAT3, which correspond to 2-*O*-monoacetylated, 3-*O*-monoacetylated and 2,3-di-*O*-acetylated Xyl residues based on the reported chemical shifts of the corresponding acetyl groups in *E*. *globulus* xylan [36]. In contrast, the predominant resonance signals in the reaction products of PtrXOAT11 and PtrXOAT12 were attributed to 2-*O*-monoacetylated and 3-*O*-monoacetylated Xyl residues (Fig 5, left panel). It was noted that the relative ratios between the resonance signals for 2-*O*- and 3-*O*-monoacetylated Xyl residues varied among the reaction products of different PtrXOATs (Fig 5, left panel), indicating that PtrXOATs may have differential preference in acetylation at *O*-2 and *O*-3. The recombinant PtrXOAT proteins were further examined for their acetyltransferase activities using the GlcA-substituted xylotetraose acceptor. It was found that the reaction products of only two of them, PtrXOAT9 and PtrXOAT10, exhibited a resonance peak that corresponded to 3-*O*-acetylated 2-*O*-GlcA-substituted Xyl residues (Fig 5, right panel). No resonance peaks corresponding to 2-*O*-monoacetylated, 3-*O*-monoacetylated and 2,3-di-*O*-acetylated Xyl residues were observed in the reaction products of PtrXOATs (Fig 5, right panel), including PtrXOAT1, PtrXOAT2, PtrXOAT3, PtrXOAT11 and PtrXOAT12 that readily transferred acetyl groups onto the unsubstituted xylotetraose acceptor (Fig 4C). The inability of these PtrXOATs to acetylate the unsubstituted Xyl residues in the GlcA-substituted xylotetraose acceptor indicates that the GlcA substitution might cause steric hindrance preventing these PtrXOATs from accessing the unsubstitued Xyl residues in the oligomer. These structural analyses of PtrXOAT-catalyzed reaction products demonstrate that PtrXOATs are xylan acetyltransferases with differential regiospecificity.

**Fig 6:**
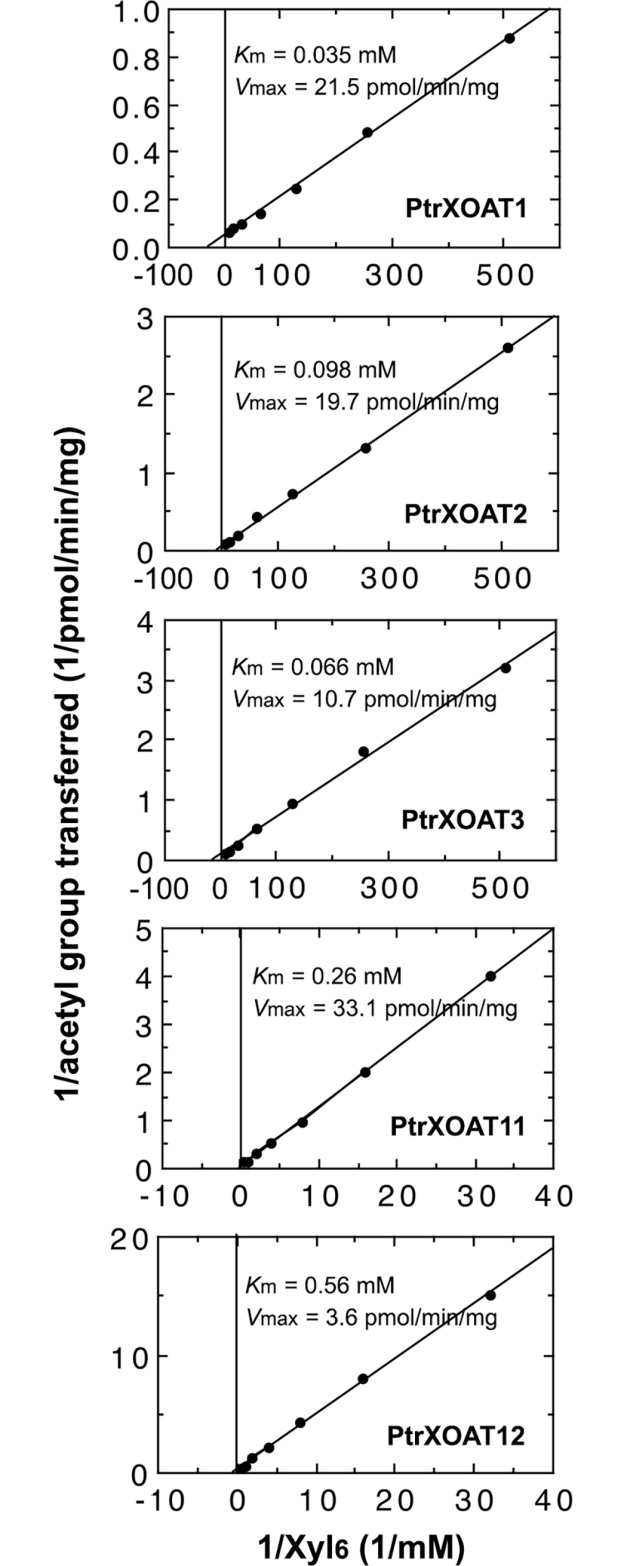
Kinetic properties of the acetyltransferase activities of PtrXOATs. Recombinant PtrXOATs were assayed for their xylan acetyltransferase activities by incubation with ^14^C-acetyl CoA and various concentrations of Xyl_6_. Lineweaver-Burk plots were used to calculate *V*_max_ and *K*_m_ values.

To decipher their biochemical properties, we further examined the acetyltransferase activities of PtrXOAT1, PtrXOAT2, PtrXOAT3, PtrXOAT11 and PtrXOAT12, which exhibited the highest activity toward the xylohexaose acceptor as shown in Fig 4B, by incubating them with different concentrations of xylohexaose (Fig 6). Analysis of their specific acetyltransferase activities using Lineweaver-Burk plots revealed that the *K*_m_ values were 0.035, 0.098, 0.066, 0.26 and 0.56 mM and the *V*_max_ values were 21.5, 19.7, 10.7, 33.1 and 3.6 pmol/min/mg protein for PtrXOAT1, PtrXOAT2, PtrXOAT3, PtrXOAT11 and PtrXOAT12, respectively. These results indicate that PtrXOAT1, PtrXOAT2 and PtrXOAT3 have a much higher affinity for the xylohexaose acceptor than PtrXOAT11 and PtrXOAT12.

### Expression of PtrXOAT1, PtrXOAT2 and PtrXOAT3 is able to partially rescue the xylan acetylation defects of the Arabidopsis *esk1* mutant

**Fig 7:**
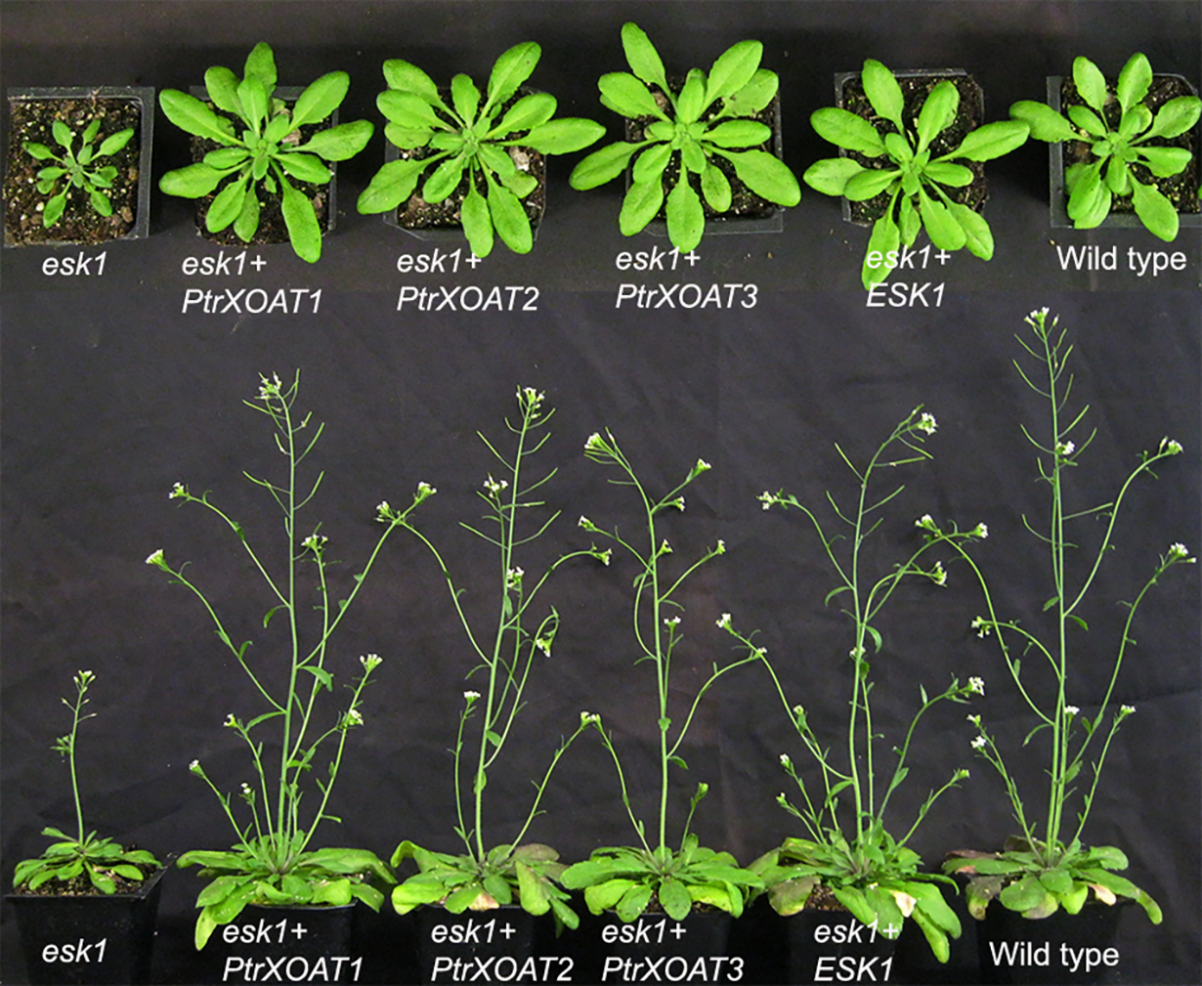
Complementation of the *esk1* mutant by PtrXOATs. Full-length cDNAs of PtrXOATs driven by the *CesA7* promoter were introduced into *esk1* mutant plants and first-generation transgenic plants were examined for their growth. Note the restoration of rosette size (top panel) and inflorescence height (bottom panel) in PtrXOAT-complemented *esk1* plants.

**Fig 8:**
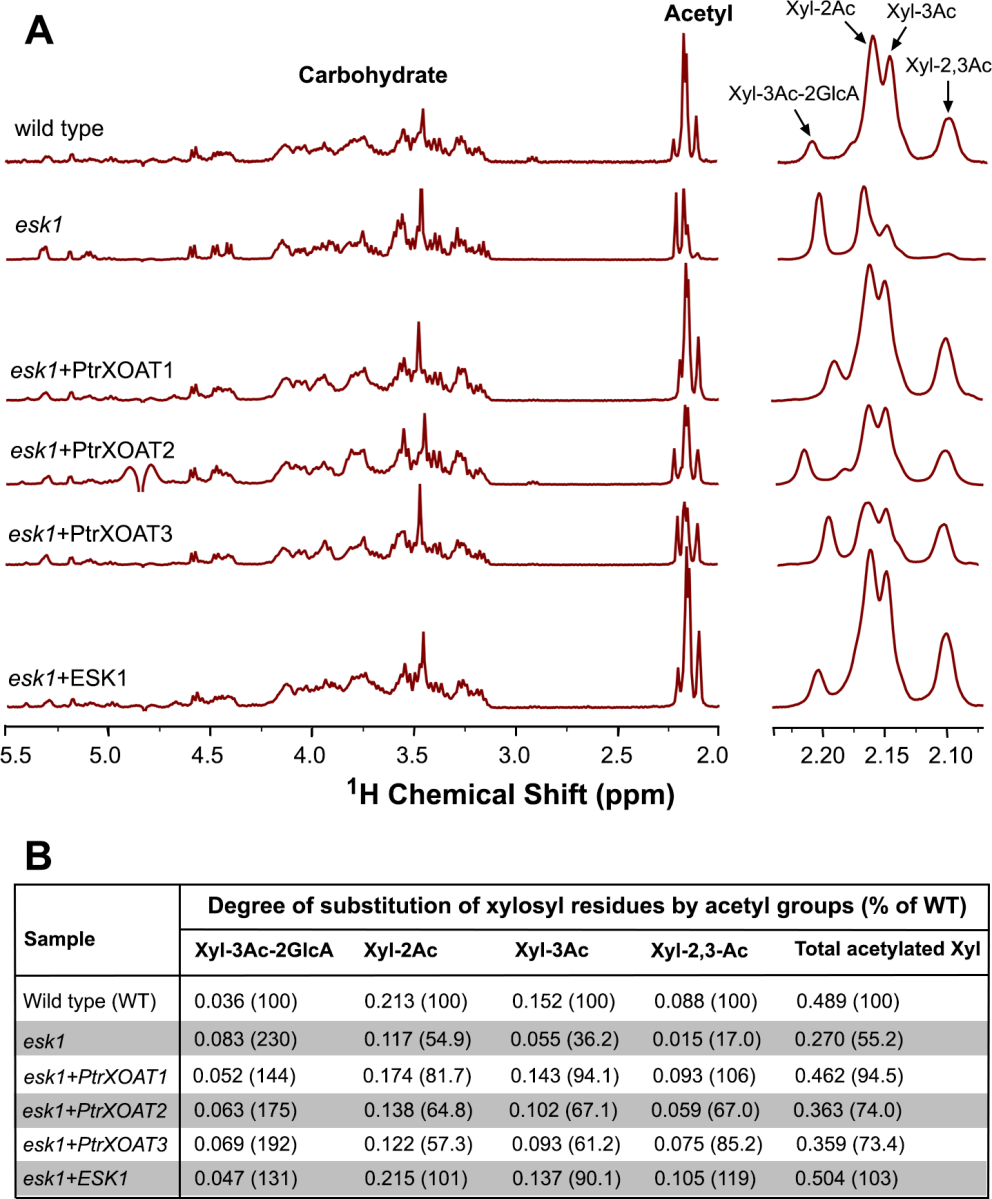
^1^H NMR analysis of acetyl xylooligomers from PtrXOAT-complemented *esk1* plants. DMSO-extracted acetyl xylans were digested with xylanase and the acetylation patterns of released xylooligomers were analyzed using ^1^H NMR spectroscopy. (A) ^1^H NMR spectra of acetyl xylooligomers from the wild type, *esk1*, and PtrXOAT-complemented *esk1*. Acetyl xylooligomers from ESK1-complemented *esk1* were used for comparison. The spectra in the left panel show the resonances attributed to carbohydrate (3.0–5.5 ppm) and acetyl groups (2.0–2.25 ppm). The enlarged spectra in the right panel show the resonances corresponding to acetyl groups in Xyl-2,3Ac, Xyl-3Ac, Xyl-2Ac, and Xyl-3Ac-2GlcA. There was a slight shift in the position of the resonance signal for Xyl-3Ac-2GlcA in some samples. (B) Integration analysis of the degree of acetyl substitution in xylans from the wild type, *esk1*, and PtrXOAT-complemented *esk1* based on the resonance signals for the carbohydrate and the acetyl groups in (A). The relative amount of acetyl groups (% of WT) was determined by the ratio of acetyl groups in the xylans of PtrXOAT-complemented *esk1* over that of the wild type (taken as 100).

Because PtrXOAT1, PtrXOAT2 and PtrXOAT3 exhibited the same regiospecificity of xylan acetylation as Arabidopsis ESK1 (Fig 5) [35], we investigated whether they were able to complement the xylan acetylation defects of the *esk1* mutant. Mutation of the *ESK1* gene was previously shown to result in a strong retardation of plant growth, including a small rosette size and reduced plant height [31]. Expression of PtrXOAT1, PtrXOAT2 and PtrXOAT3 under the promoter of the secondary wall-specific *cellulose synthase A catalytic subunit 7* (*CesA7*) gene in the *esk1* mutant effectively rescued the plant growth defects to a level comparable to that of the *esk1* complemented by ESK1 (Fig 7). Further examination of xylan structure using ^1^H NMR spectroscopy revealed that the resonance signals corresponding to 2-*O*-monoacetylation, 3-*O*-monacetylation and 2,3-di-*O*-acetylation were all increased in xylans from transgenic *esk1* expressing PtrXOAT1, PtrXOAT2, PtrXOAT3 or ESK1 compared with the *esk1* mutant (Fig 8A and 8B). Although the total degree of xylan acetylation in *esk1* was reduced to 55.2% of that of the wild type, expression of PtrXOAT1, PtrXOAT2, PtrXOAT3 and ESK1 in *esk1* restored the degree of xylan acetylation to 94.5%, 74.0%, 73.4% and 103%, respectively, of that of the wild type. These complementation analyses further corroborate that PtrXOAT1, PtrXOAT2 and PtrXOAT3 are xylan acetyltransferases catalyzing 2-*O*-monacetylation, 3-*O*-monacetylation and 2,3-di-*O*-acetylation, which is similar to ESK1. A slight shift in the position of the resonance signal for 3-*O*-acetylated 2-*O*-GlcA-substituted Xyl residues was noted in some samples (Fig 8A, right panel), which is likely attributed to the high sensitivity of the methyl protons of the 3-*O*-acetyl group to a slight variation of pH because of its close proximity to the *O*-2-linked acidic GlcA residue.

## Discussion

*Populus* is considered a promising feedstock crop for cellulosic biofuel production. One of the obstacles for biofuel production is biomass recalcitrance, i.e., resistance of biomass to enzymatic conversion into fermentable sugars, and acetylation in xylan is one factor contributing to biomass recalcitrance [38]. In this report, we have demonstrated that twelve *P*. *trichocarpa* DUF231-containing proteins are xylan acetyltransferases and among them, several are preferentially expressed in stems undergoing secondary growth and hence likely play major roles in xylan acetylation during wood formation. Our findings not only further our understanding of the biochemical mechanisms controlling the acetylation of xylan, one of the major biopolymers in wood, but also provide new molecular tools for genetic manipulation of xylan acetylation in tree species.

Our comprehensive biochemical and structural studies have revealed that the twelve PtrXOATs are xylan acetyltransferases with differential activities and among them, PtrXOAT1, PtrXOAT2, PtrXOAT3, PtrXOAT11 and PtrXOAT12 have the highest activity catalyzing the transfer of acetyl groups onto xylooligomer acceptors (Fig 4). These PtrXOATs were found to exhibit positional preference in the transfer of acetyl groups onto Xyl residues, e.g., PtrXOAT1, PtrXOAT2 and PtrXOAT3 catalyze 2-*O*- and 3-*O*-monoacetylation and 2,3-di-*O*-acetylation of Xyl residues, PtrXOAT11 and PtrXOAT12 catalyze 2-*O*- and 3-*O*-monoacetylation of Xyl residues, and PtrXOAT9 and PtrXOAT10 mediate 3-*O*-acetylation of 2-*O*-GlcA-substituted Xyl residues (Fig 5). These findings indicate that these PtrXOATs may be involved in acetylation at different positions of Xyl residues in *Populus* xylan. Most of PtrXOATs apparently share the same regiospecificity as their closest Arabidopsis homologs. For example, PtrXOAT1, PtrXOAT2, PtrXOAT3 and their closest Arabidopsis homologs, ESK1 and TBL28, all catalyze 2-*O*- and 3-*O*-monoacetylation and 2,3-di-*O*-acetylation of Xyl residues (Fig 5) [35], which is further confirmed by complementation analysis showing that PtrXOAT1, PtrXOAT2 and PtrXOAT3 are able to rescue the defects in xylan acetylation in the *esk1* mutant (Fig 8). Furthermore, PtrXOAT9, PtrXOAT10 and their closest Arabidopsis homologs, TBL32 and TBL33, appear to be the only acetyltransferases capable of mediating 3-*O*-acetylation of 2-*O*-GlcA-substituted Xyl residues (Fig 5) [35]. However, PtrXOAT11 and PtrXOAT12, which catalyze 2-*O*- and 3-*O*-monoacetylation but not 2,3-di-*O*-acetylation of Xyl residues, appear to differ from their closest Arabidopsis homologs, TBL34 and TBL35, which are able to mediate 2,3-di-*O*-acetylation of Xyl residues in addition to 2-*O*- and 3-*O*-monoacetylation (Fig 5) [35]. Therefore, although the overall regiospecificity of most PtrXOATs and their Arabidopsis homologs are conserved, some of them have diverged to have differential specificities.

PtrXOATs are predicted to have a very short N-terminal cytoplasmic tail, one transmembrane helix and a Golgi lumen-localized catalytic domain (S1 Fig). The recombinant PtrXOATs used in this study contain all amino acids right after the transmembrane helix and thus harbor the entire catalytic domain. Therefore, they should retain the activity of the full-length protein although the possibility that other modifications, such as C-terminal tagging, may affect their activity could not be excluded. Since full-length recombinant PtrXOATs would be Golgi-localized, their purification requires detergent for solubilization of membrane proteins, which might have a detrimental effect on their enzymatic activity. The secreted soluble form of recombinant PtrXOATs currently represents the best option for studying their xylan acetyltransferase activity. The fact that the regiospecific activities of recombinant Arabidopsis xylan acetyltransferases including ESK1 and its homologs are in agreement with the corresponding defects in xylan acetylation caused by their mutations [35] indicates that removal of the N-terminal transmembrane domain and addition of a C-terminal tag in the recombinant proteins do not alter their specificities.

The degree of xylan acetylation at different positions of Xyl residues was also found to vary between *Populus* stems and Arabidopsis stems. While the degree of monoacetylation at *O*-2 or *O*-3 is similar (Fig 1) [35], that of 2,3-di-*O*-acetylation is much higher in *Populus* than in Arabidopsis. In addition, of the 2-*O*-GlcA-substituted Xyl residues that constitute about 10% of the total Xyl residues in xylans of both *Populus* and Arabidopsis [22,28], about 40% are acetylated at *O*-3 in Arabidopsis [28,35] but nearly all of them are acetylated at *O*-3 in *Populus* (Fig 1). The observed difference in the degree of xylan acetylation at different positions between *Populus* and Arabidopsis could be due to differences in the activities and/or affinities of PtrXOATs and their Arabidopsis counterparts.

Although it is evident that PtrXOATs display differential positional preference in 2-*O*- and 3-*O*-monoacetylation (Fig 5), it should be cautioned that spontaneous migration of acetyl groups between *O*-2 and *O*-3 on a Xyl residue might occur. It is currently challenging to discern whether spontaneous migration of acetyl groups might have contributed to the observed difference in the positional preference of PtrXOATs. A previous study has suggested that ESK1 catalyzes 2-*O*-monoacetylation of Xyl residues and the acetyl groups at *O*-3 are spontaneously migrated from *O*-2 based on the observation that 3-*O*-monoacetylation had a lag time of 30 min compared with 2-*O*-monoacetylation [32]. However, this slight lag of 3-*O*-monoacetylation could be due to the differential affinity of ESK1 toward the *O*-2 and *O*-3 positions of Xyl residues for acetylation. The fact that ESK1 and its close *Populus* homologs PtrXOAT1, PtrXOAT2 and PtrXOAT are also able to mediate xylan 2,3-di-*O*-acetylation indicates that they possess both 2-*O*- and 3-*O*-acetyltransferase activities. It remains to be determined whether they acetylate at *O*-2 of Xyl first and then at *O*-3 of Xyl that is already acetylated at *O*-2 or vice versa.

Among the 12 PtrXOAT genes, *PtrXOAT1*, *PtrXOAT2*, *PtrXOAT9* and *PtrXOAT10* showed preferential expression in *Populus* stems undergoing secondary growth, indicating that they are likely the major acetyltransferases involved in xylan acetylation during wood formation. The expression of PtrXOAT1, PtrXOAT2, PtrXOAT9 and PtrXOAT10 was shown to be activated by the wood-associated secondary wall NAC master regulator, PtrWND2B, which further corroborates their involvement in wood formation. Since PtrXOAT1 and PtrXOAT2 catalyze 2-*O*- and 3-*O*-monoacetylation and 2,3-di-*O*-acetylation and PtrXOAT9 and PtrXOAT10 mediate 3-*O*-acetylation of 2-*O*-GlcA-substituted Xyl residues, the combined activities of these four PtrXOATs could contribute predominantly to xylan acetylation during wood formation in *Populus*.

PtrXOATs belong to a family of DUF231-containing proteins with a TBL domain and a DUF231 domain. The two conserved motifs, the GDS motif and the DXXH motif, residing in the TBL domain and the DUF231 domain, respectively, were first identified in Arabidopsis DUF231 proteins [39]. The serine residue in the GDS motif and the histidine and aspartate residues in the DXXH motif of ESK1 have been shown to be essential for its acetyltransferase activity and therefore it was proposed that xylan acetyltransferases may have adopted the Ser-His-Asp catalytic triad for their mechanism of action [35], which is similar to that for serine esterases and proteases [40]. All 12 PtrXOATs have the conserved GDS motif and DXXH motif in the TBL domain and the DUF231 domain, respectively (S2 Fig), indicating an essential role of these motifs in their acetyltransferase activities. In addition to the 12 PtrXOATs, there exist 52 additional DUF231 genes in the genome of *P*. *trichocarpa* (Fig 2) and their biochemical functions remain to be investigated. Among the 46 DUF231 proteins in Arabidopsis, only the 9 XOATs and two other members, AXY4 and AXY4L, have been shown to be involved in polysaccharide acetylation. Mutations of AXY4 and AXY4L have been demonstrated to cause a defect in acetylation of galactose residues in xyloglucan, a major hemicellulose in primary walls [41]. Because acetylation of biopolymers in plant biomass is one of the major factors contributing to biomass recalcitrance for biofuel production, further investigation of roles of *Populus* DUF231 proteins in polysaccharide acetylation will help generate knowledge that could provide a basis for custom-designing wood polymer composition better suited for biofuel production.

## Methods

### Cell wall isolation

Stems of *P*. *trichocarpa* and *A*. *thaliana* were ground into powder in liquid N_2_, which was then homogenized sequentially in 70% ethanol, 100% ethanol, and 100% acetone to generate alcohol-insoluble cell wall residues [42]. Acetyl xylan was extracted with dimethyl sulfoxide (DMSO) from the cell wall residues according to Evtuguin *et al*. [24]. DMSO has been widely used to extract acetyl xylans from both gymnosperm and angiosperm wood [23–25]. Briefly, cell walls were delignified with 10% peracetic acid to obtain holocellulose. The holocellulose was then extracted with 100% DMSO to release acetyl xylan. After extraction with DMSO for four times, little xylose remained in the holocellulose as detected by cell wall composition analysis (0 mg Xyl/g holocellulose), indicating that DMSO efficiently released xylan from the holocellulose. Acetyl xylooligomers were generated by incubating acetyl xylan with β-endoxylanase M6 (Megazyme) at 40°C for 12 hr according to Megazyme’s protocol.

### ^1^H NMR spectroscopy

^1^H NMR spectra of acetyl xylooligomers were obtained with a Bruker Avance III HD 400 MHz spectrometer. The NMR spectra were recorded with 512 transients and the resonance signals for acetyl groups at different positions of Xyl residues were assigned according to the reported NMR spectra data for *Eucalyptus globulus* xylan [36]. Xylooligomers from three biological samples were examined and representative NMR spectra were shown.

### Gene expression analysis

Total RNA was isolated from *P*. *trichocarpa* tissues using the Qiagen RNeasy Plant mini kit (Qiagen). The tissues used were expanding leaves and their petioles, stem segments with primary growth (no secondary xylem was evident by examining their cross sections) (stem-I), and stem segments with secondary growth (secondary xylem was evident by examining their cross sections) (stem-II). The DNase-treated RNA was used for real-time quantitative PCR analysis. The primers used for PCR analysis were 5’- aggctctttgtggttgcagctaat-3’ and 5’-caaaacattgcacgtgttaattcc-3’ for *PtrXOAT1*, and 5’- aggctttttgcggttgcagccaac-3’ and 5’-gcctcgcacgtgttaattcctatc-3’ for *PtrXOAT2*, 5’-gtttacttgcttaacatcaccacc-3’ and 5’-acacctttggagaaagcaatattc-3’ for *PtrXOAT3*, 5’-ggagggtagcaatgaccatcgata-3’ and 5’-tcaatattggttggtgatgtatgc-3’ for *PtrXOAT4*, 5’-ccagtggatgtgagcacagacagg-3’ and 5’-caagagagtgctatgattcgtgca-3’ for *PtrXOAT5*, 5’-ccattcgatgtgggaacgaaccga-3’ and 5’-caagtacgagagatgatatatgtg-3’ for *PtrXOAT6*, 5’-gatgagtgtggtggccagcatagg-3’ and 5’-ctacaaataagcaaggaatatccg-3’ for *PtrXOAT7*, 5’- gttgatgatatcctacgagaatcg-3’ and 5’-taacaaatatgcgtagagaatctc-3’ for *PtrXOAT8*, 5’-gtttcaatgaaactactttgataa-3’ and 5’-acaggtttcacttgcgagtggatg-3’ for *PtrXOAT9*, 5’-gttacaacgaaacaactctagttg-3’ and 5’-attcatggcttgctagtgtatgtg-3’ for *PtrXOAT10*, 5’-agaggattaaatgtacaaatgatt-3’ and 5’-ctgtcaaagattaataatgtgagc-3’ for *PtrXOAT11*, and 5’-ttgggatcaaaggtttcagttctc-3’ and 5’-tgcaatgtggacaggtacatttgc-3’ for *PtrXOAT12*. The primers used for PCR analysis were designed to specifically amplify the gene of interest and their specificities were further verified by sequencing the PCR-amplified products (see the deposited sequences at GenBank with the accession numbers described below). The copy numbers of transcripts were calculated based on the PCR threshold cycle numbers of the samples and the standard curves of plasmid controls. For each sample, three biological replicates were analyzed. Transgenic *Populus* plants with PtrWND2B overexpression were generated previously [37] and the seedling leaves were used for RNA isolation and subsequent expression analysis. The expression level of a *Populus* actin gene (*PtrACT7*; Potri.019G010400) was used for normalization among different samples.

### Recombinant PtrXOAT production

The cDNA sequences of PtrXOATs with deletion of the N-terminal transmembrane domain were cloned into the pSecTag2 mammalian expression vector (Invitrogen) such that the murine Igκ chain leader sequence (for protein secretion) was at the amino terminus of PtrXOATs and the c-myc epitope and the six tandem histidine tag were at the carboxyl terminus of PtrXOATs. The recombinant PtrXOAT proteins thus generated include the amino acid sequence from 44 to 490 for PtrXOAT1, 44 to 487 for PtrXOAT2, 35 to 418 for PtrXOAT3, 37 to 428 for PtrXOAT4, 33 to 470 for PtrXOAT5, 36 to 446 for PtrXOAT6, 38 to 426 for PtrXOAT7, 33 to 415 for PtrXOAT8, 52 to 437 for PtrXOAT9, 55 to 438 for PtrXOAT10, 36 to 409 for PtrXOAT11 and 30 to 447 for PtrXOAT12. For production of recombinant PtrXOAT proteins, human embryonic kidney (HEK) cells 293F were transfected with the expression constructs using the Invitrogen FreeStyle 293 Expression System [35]. The culture medium was collected and passed through a nickel resin column. After extensive washing, recombinant PtrXOAT proteins were eluted from the resin with the elution buffer [10 mM Tris-HCl (pH 7.0), 50 mM KCl and 300 mM imidazole]. The purified recombinant proteins were examined by SDS polyacrylamide gel electrophoresis and Coomassie Blue staining.

### Acetyltransferase activity assay

The recombinant PtrXOAT proteins were assayed for acetyltransferase activity using ^14^C-acetyl CoA as the acetyl donor and xylooligomers as the acceptor [35]. The reaction mixture contained 50 mM HEPES buffer, pH 7.0, acetyl-1,2-^14^C CoA (0.1 mM; American Radiolabeled Chemicals) or non-radiolabeled acetyl CoA (1 mM), xylohexaose (Xyl_6_) or GlcA-substituted xylotetraose [(GlcA)Xyl_4_] (30 μg for radioactivity-based assay and 200 μg for NMR analysis), and purified recombinant proteins (20 μg for radioactivity-based assay and 200 μg for NMR analysis). Xylooligomers were purchased from Megazyme and GlcA-substituted xylotetraose was prepared by xylanase digestion of the Arabidopsis *gxm1/2/3* xylan [43,44]. The reaction mixture was incubated at 37 ^o^C for 4 hr. For radioactivity-based assay, the reaction products were passed through Dowex 1X4 anion exchange resin to separate the radiolabeled xylooligomer products from acetyl-1,2-^14^C CoA and then counted for the amount of radioactivity with a Perkin Elmer scintillation counter. For NMR analysis, products in the reactions with xylohexaose were passed through Dowex 1X4 resin to remove acetyl CoA, and those in the reactions with (GlcA)Xyl_4_ were not due to the negative charge of GlcA residues. Recombinant PtrXOATs were also incubated with ^14^C-acetyl CoA and mannohexaose (Megazyme) or xyloglucan oligomers and their reaction products were examined for incorporation of radioactivity as described above. Xyloglucan oligomers were prepared by endo-1,4-β-glucanase digestion of wild-type Arabidopsis xyloglucan [45]. For each PtrXOAT, recombinant proteins purified from three separately transfected cultures were used for acetyltransferase activity assay and subsequent analyses.

### Complementation analysis

Full-length PtrXOAT cDNAs were ligated between the Arabidopsis *CesA7* promoter and the nopaline synthase terminator in a binary vector and the expression constructs thus created were introduced into the Arabidopsis *esk1* mutant plants by Agrobacterium-mediated transformation. Over 100 independent first-generation transgenic *esk1* plants for each PtrXOAT construct were used for analyses. To obtain sufficient cell wall materials for xylan structural analysis, stems pooled from 30 independent first-generation transgenic plants were used as one biological replicate. Three biological replicates for each construct were analyzed for xylan structure.

### Statistical analysis

The data of quantitative RT-PCR analysis and the acetyltransferase assays were analyzed using the Student’s *t* test program (http://www.graphpad.com/quickcalcs/ttest1.cfm), and the reported differences between the control and the experimental samples were found to be statistically significant (p < 0.001).

### Accession numbers

The *P*. *tricocarpa* gene locus identifiers and GenBank accession numbers for PtrXOAT genes are Potri.008G069900 and MG938528 (PtrXOAT1), Potri.010G187600 and MG938529 (PtrXOAT2), Potri.010G187500 and MG9385230 (PtrXOAT3), Potri.008G070200 and MG938531 (PtrXOAT4), Potri.010G187300 and MG938532 (PtrXOAT5), Potri.008G070000 and MG938533 (PtrXOAT6), Potri.016G119100 and MG938534 (PtrXOAT7), Potri.001G376700 and MG938535 (PtrXOAT8), Potri.008G073300 and MG938536 (PtrXOAT9), Potri.010G184000 and MG938537 (PtrXOAT10), Potri.016G125500 and MG938538 (PtrXOAT11), and Potri.016G125600 and MG938539 (PtrXOAT12), respectively.

## Supporting information

S1 Fig PtrXOATs are membrane proteins with one transmembrane helix at the N-terminus. PtrXOATs were predicted for transmembrane helices by the TMHMM2.0 program (http://www.cbs.dtu.dk/services/TMHMM/) except for PtrXOAT4 that was predicted by the TMMOD program (http://liao.cis.udel.edu/website/servers/TMMOD/scripts/frame.php?p=submit). Inside, the cytoplasmic side of the membrane; outside, the Golgi lumen side of the membrane.
(PDF)

S2 Fig Amino acid sequence alignment of the 12 *Populus trichocarpa* PtrXOATs and Arabidopsis ESK1. Shown on the left are the names of each protein, and shown on the right are the positions of the last amino acid residue in that line for each protein. Identical amino acid residues among all the members are denoted by an asterisk under the aligned sequences, amino acids with strong conservation are indicated with a colon, and those with weak conservation are marked with a period. The TBL and DUF231 domains are underlined and the GDS and DXXH motifs are boxed.
(PDF)
