# Supplementary figures and images for: A group of Populus trichocarpa DUF231 proteins exhibit differential O-acetyltransferase activities toward xylan
Source: PLoS One. 2018 Apr 4;13(4):e0194532. doi: 10.1371/journal.pone.0194532 (PMC5884507; doi:10.1371/journal.pone.0194532)

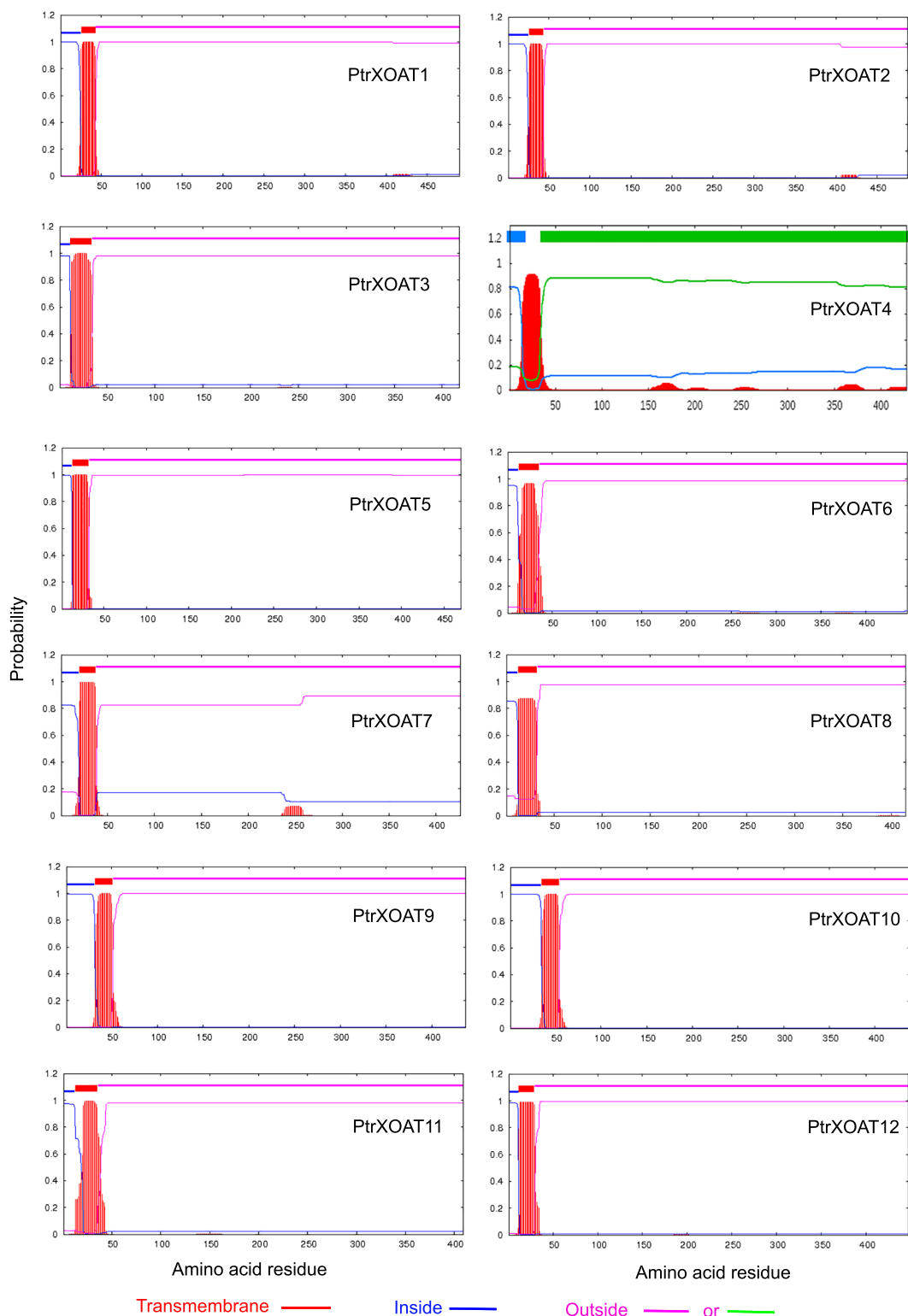

S1 Fig

Supplement: S1 Fig — PtrXOATs were predicted for transmembrane helices by the TMHMM2.0 program (http://www.cbs.dtu.dk/services/TMHMM/) except for PtrXOAT4 that was predicted by the TMMOD program (http://liao.cis.udel.edu/website/servers/TMMOD/scripts/frame.php?p=submit). Inside, the cytoplasmic side of the membrane; outside, the Golgi lumen side of the membrane. (PDF) [file pone.0194532.s001.pdf]
