# Supplementary material for: A group of Populus trichocarpa DUF231 proteins exhibit differential O-acetyltransferase activities toward xylan
Source: PLoS One. 2018 Apr 4;13(4):e0194532. doi: 10.1371/journal.pone.0194532 (PMC5884507; doi:10.1371/journal.pone.0194532)
Supplement: S2 Fig — Shown on the left are the names of each protein, and shown on the right are the positions of the last amino acid residue in that line for each protein. Identical amino acid residues among all the members are denoted by an asterisk under the aligned sequences, amino acids with strong conservation are indicated with a colon, and those with weak conservation are marked with a period. The TBL and DUF231 domains are underlined and the GDS and DXXH motifs are boxed. (PDF) [file pone.0194532.s002.pdf]

```
PtrXOAT8        -----------MTIKPS-------------LDRRIQT---LFPVALACLLVLG------TA      28
PtrXOAT7        -----------MSLAPS---------QSAAMKPSKGKLPISVVTIIICSFAFIALLYTDTS      41
PtrXOAT4        ----------------------MKQGKFGNLKIQHSKLVSSTVA-IFLISLLPCFVFSEHA      38
PtrXOAT5        ------------------------MKPVSGTNRRCQSFPILIVT-LFFLFFLGFFLYNDSM      36
PtrXOAT6        ------------------------MKDGGGLRSQSCNYPSI-LV-FFTFSIVACVLFNENF      35
PtrXOAT3        -----------MSLTPCRSNYK-----------WSTYYHYFLMM-VFLLLLFKIFMYSENF      38
ESK1            -------------MQPWRRKFPLFE-TGVTMKQRKNSNLSIFVV-VFSVFLFGIFMYNEDV      46
PtrXOAT1        -------------MQTSRRKSPLLSSVTIAMKHRKNNNLSVFVV-VSSIFIFGVFMYNEDV      47
PtrXOAT2        -------------MQPSRRKSPLLSSVTVTMKHRKNSNLSVFVV-VFSVFLFGVFMYNEDV      47
PtrXOAT9        MNIKKRNLKLTNMKQPFTASS-----SSSSLIRKARLSPY--LFTLLAFIVFVAILYGEDF      54
PtrXOAT10       MN-IKRSLKFSKMKQPFTASSSS-SSSSSSLIRKARLSPY--LLTLLVFIVFVAILYGEDF      57
PtrXOAT11       ------------------------------------------------------------      0
PtrXOAT12       ------------MMQRW--------------HRKKSHFPL--VAFIFLGIIVCSILYNESS      33

PtrXOAT8        RLV---------------------------------------LDSLKSNQ------------      41
PtrXOAT7        RSLFKLKSC--------------------PRR-----------------------------      55
PtrXOAT4        DSGSIPSHL-------------------------------------KSDKSHHRTERK----      61
PtrXOAT5        KFSA---FQVPRPPSHQEMLREPVNSKVPTVQETPSRNKH--------------DRDRNAE      80
PtrXOAT6        VALP---FP--RFKGILEEYPAV--------QENSNIDD----------------------      63
PtrXOAT3        KSVFSGSFLLS-------------------------------------------------      51
ESK1            KSIAEFPFSTSKPHDVHDEATPITEITTLPVQESIKNSDPIQESIKNADSVQDSVKDVA--      107
PtrXOAT1        KSIAEFPFSWPKSQEIQEELSK----GTTPVQETLKKDRELPASVGSRTSLEEPQVDQEFE      104
PtrXOAT2        KSIAEFPFSWPKSQE---EPSK----GVTPVQETLEKDQELPASVGSRTSLEEPQVDQGPG      101
PtrXOAT9        VCLLGQLDPNVDRL----------------------------------------------      70
PtrXOAT10       MCLLGQLDPNLDRP----------------------------------------------      73
PtrXOAT11       -----------------MATTKQ-----LM----------VPATWGIRSSFHSLIALL---      28
PtrXOAT12       IQQVHEEDPSNQGPNHQQHATTV-----TY----------VKPNLGTHSNFAPVLL-----      76

PtrXOAT8        -----SSIFRVYGRQEGGEYKKPVFVLP-------EDRFEKGCDVFEGQWVWDNVSRPLYT      88
PtrXOAT7        -----NPVKKSKDRTAE--DK-----LKNFQMDDRFEFDPEECTVNTGKWMFNKTLKPLYT      102
PtrXOAT4        ---QE---------------DKEVF-VSTKQVEKLRSEPKDSCDIFTGKWVFDNKTHPLYR      101
PtrXOAT5        GGEDGDNQIINLSMTLGG-PKMELPAVEKEDADEKIALQPEECDIFTGHWVLDNKTHPLYK      140
PtrXOAT6        -------VRPSAKGTKEN-VNMELVSVEGDHEDEEVVLPREECDIFLGEWVLDKLTHPLYK      114
PtrXOAT3        --------------------RNEVH--GIREKDEEVILPPKDCDLFTGKWVFDNATHPLYK      88
ESK1            -----EPVQEEVSKTEEV-KKIELF--AATEDEEDVELPPEECDLFTGEWVFDNETHPLYK      158
PtrXOAT1        EN-QESDKLKSSGSKEDE-EKIELP--IIEEDDVDVELPPEECDLFTGEWVFDNETRPLYK      161
PtrXOAT2        EV-QESDNLKSSESKEDE-EKIEFP--VIEEDDEDVELPPEECDLFTGQWVFDNETRPLYK      158
PtrXOAT9        -----------ATITGELKKR-----WE--KLPFSIGKAPDGCDLFSGRWVWDDSTRPLYE      111
PtrXOAT10       -----------ATS--RTEKK-----WE--KLPFSTGRTPEGCDFFSGRWVWDDSNRPLYE      112
PtrXOAT11       ---VAFLVIASIYVTQNSGVL-----VEDRTKSKSSGDLLSRCNLFSGKWVFDNKSYPLYK      79
PtrXOAT12       ---DRFSRCNSTSEYSGKRIR-----WGDSKVERGRRKSLESCDVFAGKWVFDRESYPLYN      127
                                                       . * .  *.*: :    ***

PtrXOAT8        EESCPYLVKQTTCQRNGRPDSFYQDWRWQPHACKLPRFDPLKLLDVLRGKRLMFIGDSVQR      149
PtrXOAT7        DRSCPYLDRQVSCVKNGRRDSYRRWEWQPDDCTLPRFNPELALKKLRGKRLLFVGDSLQR      163
PtrXOAT4        EDECPYIQQWISCTKNGRPDSMYQSWRWQPKGCSLPKFNAKLFLEKLRGKRLMFVGDSIHQ      162
PtrXOAT5        EDGCEFLSEWVRCLRNGRRDSLYQNWRWQPRDCSLPKFEPKLLLEKLKGKRLMFVGDSIHF      201
PtrXOAT6        EEDCEFLTDSVTCIKNGRKDSMYQNWRWQPRDCSLPKFKATLLLEKLRGKRLMFVGDSLNR      175
PtrXOAT3        EEECEFLSRQVTCLRNGRQDSLYQNWRWQPRDCSLPRFKAKLLLEKLRGKRLMFVGDSLNR      149
ESK1            EDQCEFLTAQVTCMRNGRRDSLYQNWRWQPRDCSLPKFKAKLLLEKLRNKRMMFVGDSLNR      219
PtrXOAT1        EDECEFLTAQVTCMRNGRKDSLYQNWKWQPRDCSLPKFKPRLLLNKLRNKRLMFVGDSLNR      222
PtrXOAT2        EDECEFLTAQVTCMRNGRKDSLYQNWKWQPRDCSLPKFKPRLLLNKLRNKRLMFVGDSLNR      219
PtrXOAT9        ESECPYIQPQLTCQEHGRPDKDYQYWRWQPYGCDLPSFNATLMLETLRGKRMMFVGDSLNR      172
PtrXOAT10       ESECPYIQPQLTCQEHGRPEKDYQHWRWQPHGCDLPSFNATLMLETLRGKRMMFVGDSLNR      173
PtrXOAT11       EKECTFMSDQLACEKFGRKDLNYQNWRWQPHQCDLPRFNATVLLERLRNRRLMFVGDSLNR      140
PtrXOAT12       ESDCPYMSDQLACHKHGRSDLGYQHWRWQPHDCNLKRWNVTEMWEKLRGKRLMFVGDSLNR      188
                :  * ::     * . ** :  *: *.*** * *  :.     . *:.:*::*:***::

PtrXOAT8        GQFESMVCMVQSVIPDGKKSFHR--IP-PMKIFKAEEYNASIEYYWAPFIVESISDHA-TN      207
PtrXOAT7        GQWQSFVCLVEWIIPEDKKSMKE--VG-SHSVFRAKEYDATIEFYWAPFLIESNTDHH-II      221
PtrXOAT4        NQWMSLVCLVQSAISPGKKRTTF--ST-YSNRFIIEEYNATIESYWAPFLVKSNGDPPKMR      220
PtrXOAT5        NQWQSLICLVQSAIPPGKKSLDY--AS-YITVFKIEDYNATLEFYWAPFLVESNSDPPTMR      259
PtrXOAT6        QQWESMICLVQSVIPLDKKSLSS--SSSFLSVFKIEDYNATIEFYWAPFLVESNSDAVSNR      234
PtrXOAT3        NQWESMVCLLQSGAPLAKKSLSD--SG-SASFFRIEDYNTTVEFYWAPFLVESNSDHP-SK      207
ESK1            NQWESMVCLVQSVVPPGRKSLNK--TG-SLSVFRVEDYNATVEFYWAPFLVESNSDDP-NM      277
PtrXOAT1        NQWESMICFVQSVIPPGRKSLNK--TG-SLAVFRIEDYNATVEFYWAPFLVESNSDDP-NM      280
PtrXOAT2        NQWESMVCFVQSLIPPGRKSLNK--TG-SLAVFRIEDYNATVEFYWAPFLVESNSDDP-NM      277
PtrXOAT9        GQYVSMVCLLHRLIPEGQKSMET--FD-SLTVFTAKEYNATIEFYWAPFLLESNSDNA-IV      230
PtrXOAT10       GQYVSMVCLLHRLIPEDQKSMET--FG-SLTVFTAKEYNATIEFYWAPFLLESNSDDA-VI      231
PtrXOAT11       GQWVSMVCLVDSVIPPGLQSMHQYRNG-SLHIYKATEYNATIEFYWSPLLVESNSDDP-VY      200
PtrXOAT12       GQWISMVCLLQSVIPADKRSMS--PNA-PLTIFRAEEYNATIEFLWAPLLVDSNSDDP-VN      246
                 *: *::*::.       :            :   :*::::*  *:*:::.*  *
```

Start of the TBL domain

GDS motif

End of the TBL domain

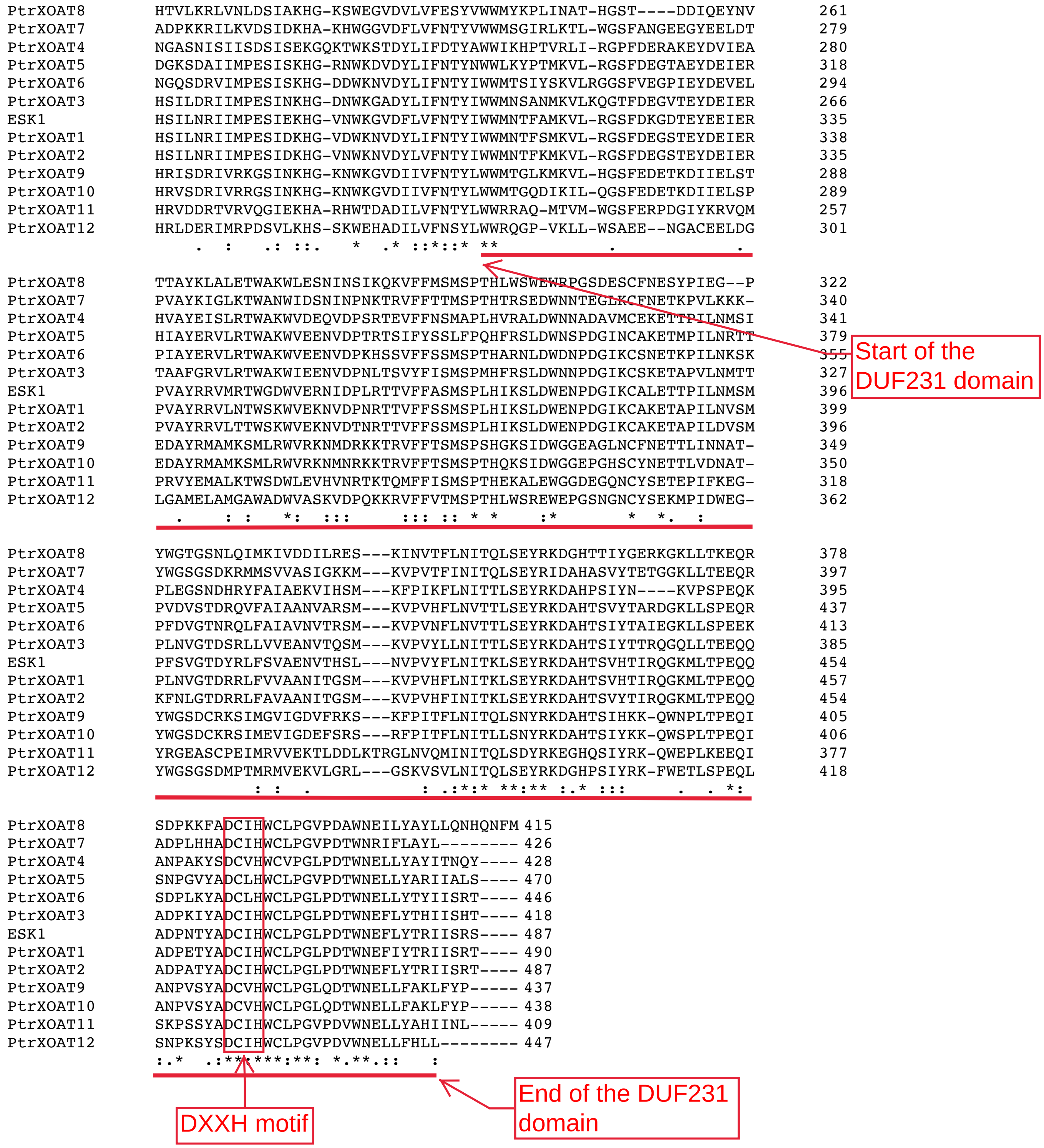
PtrXOAT8 HTVLKRLVNLDSIAKHG-KSWEGVDVLVFESYVWWMYKPLINAT-HGST----DDIQEYNV 261
PtrXOAT7 ADPKKRILKVDSIDKHA-KHWGGVDFLVFNTYVWWMSGIRLKTL-WGSFANGEEGYEELDT 279
PtrXOAT4 NGASNISIISDSISEKGQKTWKSTDYLIFDTYAWWIKHPTVRLI-RGPFDERAKEYDVIEA 280
PtrXOAT5 DGKSDAIIMPESISKHG-RNWKDVDYLIFNTYNWWLKYPTMKVL-RGSFDEGTAEYDEIER 318
PtrXOAT6 NGQSDRVIMPESISKHG-DDWKNVDYLIFNTYIWWMTSIYSKVLRGGSFVEGPIEYDEVEL 294
PtrXOAT3 HSILDRIIMPESINKHG-DNWKGADYLIFNTYIWWMNSANMKVLKQGTFDEGVTEYDEIER 266
ESK1 HSILNRIIMPESIEKHG-VNWKGVDFLVFNTYIWWMNTFAMKVL-RGSFDKGDTEYEEIER 335
PtrXOAT1 HSILNRIIMPESIDKHG-VDWKNVDYLIFNTYIWWMNTFSMKVL-RGSFDEGSTEYDEIER 338
PtrXOAT2 HSILNRIIMPESIDKHG-VNWKNVDYLVFNTYIWWMNTFKMKVL-RGSFDEGSTEYDEIER 335
PtrXOAT9 HRISDRIVRKGSINKHG-KNWKGVDIIVFNTYLWWMTGLKMKVL-HGSFEDETKDIIELST 288
PtrXOAT10 HRVSDRIVRRGSINKHG-KNWKGVDIIVFNTYLWWMTGQDIKIL-QGSFEDETKDIIELSP 289
PtrXOAT11 HRVDDRTVRVQGIEKHA-RHWTDADILVFNTYLWWRRAQ-MTVM-WGSFERPDGIYKRVQM 257
PtrXOAT12 HRLDERIMRPDSVLKHS-SKWEHADILVFNSYLWWRQGP-VKLL-WSAEE--NGACEELDG 301
. : .: ::. * .* ::*::* ** . .
PtrXOAT8 TTAYKLALETWAKWLESNINSIKQKVFFMSMSPTHLWSWEWRPGSDESCFNESYPIEG--P 322
PtrXOAT7 PVAYKIGLKTWANWIDSNINPNKTRVFFTTMSPTHTRSEDWNNTEGLKCFNETKPVLKKK- 340
PtrXOAT4 HVAYEISLRTWAKWVDEQVDPSRTEVFFNSMAPLHVRALDWNNADAVMCEKETTPILNMSI 341
PtrXOAT5 HIAYERVLRTWAKWVEENVDPTRTSIFYSSLFPQHFRSLDWNSPDGINCAKETMPILNRTT 379
PtrXOAT6 PIAYERVLRTWAKWVEENVDPKHSSVFFSSMSPTHARNLDWDNPDGIKCSNETKPILNKSK 355
PtrXOAT3 TAAFGRVLRTWAKWIEENVDPNLTSVYFISMSPMHFRSLDWNNPDGIKCSKETAPVLNMTT 327
ESK1 PVAYRRVMRTWGDWVERNIDPLRTTVFFASMSPLHIKSLDWENPDGIKCALETTPILNMSM 396
PtrXOAT1 PVAYRRVLNTWSKWVEKNVDPNRTTVFFSSMSPLHIKSLDWENPDGIKCAKETAPILNVSM 399
PtrXOAT2 PVAYRRVLTTWSKWVEKNVDTNRTTVFFSSMSPLHIKSLDWENPDGIKCAKETAPILDVSM 396
PtrXOAT9 EDAYRMAMKSMLRWVRKNMDRKKTRVFFTSMSPSHGKSIDWGGEAGLNCFNETTLINNAT- 349
PtrXOAT10 EDAYRMAMKSMLRWVRKNMNRKKTRVFFTSMSPTHQKSIDWGGEPGHSCYNETTLVDNAT- 350
PtrXOAT11 PRVYEMALKTWSDWLEVHVNRTKTQMFFISMSPTHEKALEWGGDEGQNCYSETEPIFKEG- 318
PtrXOAT12 LGAMELAMGAWADWVASKVDPQKKRVFFVTMSPTHLWSREWEPGSNGNCYSEKMPIDWEG- 362
. : : *: ::: ::: :: * * :* * *. :
PtrXOAT8 YWGTGSNLQIMKIVDDILRES---KINVTFLNITQLSEYRKDGHTTIYGERKGKLLTKEQR 378
PtrXOAT7 YWGSGSDKRMMSVVASIGKKM---KVPVTFINITQLSEYRIDAHASVYTETGGKLLTEEQR 397
PtrXOAT4 PLEGSNDHRYFAIAEKVIHSM---KFPIKFLNITTLSEYRKDAHPSIYN----KVPSPEQK 395
PtrXOAT5 PVDVSTDRQVFAIAANVARSM---KVPVHFLNVTTLSEYRKDAHTSVYTARDGKLLSPEQR 437
PtrXOAT6 PFDVGTNRQLFAIAVNVTRSM---KVPVNFLNVTTLSEYRKDAHTSIYTAIEGKLLSPEEK 413
PtrXOAT3 PLNVGTDSRLLVVEANVTQSM---KVPVYLLNITTLSEYRKDAHTSIYTTRQGQLLTEEQQ 385
ESK1 PFSVGTDYRLFSVAENVTHSL---NVPVYFLNITKLSEYRKDAHTSVHTIRQGKMLTPEQQ 454
PtrXOAT1 PLNVGTDRRLFVVAANITGSM---KVPVHFLNITKLSEYRKDAHTSVHTIRQGKMLTPEQQ 457
PtrXOAT2 KFNLGTDRRLFAVAANITGSM---KVPVHFINITKLSEYRKDAHTSVYTIRQGKMLTPEQQ 454
PtrXOAT9 YWGSDCRKSIMGVIGDVFRKS---KFPITFLNITQLSNYRKDAHTSIHKK-QWNPLTPEQI 405
PtrXOAT10 YWGSDCKRSIMEVIGDEFSRS---RFPITFLNITLLSNYRKDAHTSIYKK-QWSPLTPEQI 406
PtrXOAT11 YRGEASCPEIMRVVEKTLDDLKTRGLNVQMINITQLSDYRKEGHQSIYRK-QWEPLKEEQI 377
PtrXOAT12 YWGSGSDMPTMRMVEKVLGRL---GSKVSVLNITQLSEYRKDGHPSIYRK-FWETLSPEQL 418
: : . : .:*:* **:** :.* ::: . . *:
PtrXOAT8 SDPKKFADCIHWCLPGVPDAWNEILYAYLLQNHQNFM 415
PtrXOAT7 ADPLHHADCIHWCLPGVPDTWNRIFLAYL-------- 426
PtrXOAT4 ANPAKYSDCVHWCVPGLPDTWNELLYAYITNQY---- 428
PtrXOAT5 SNPGVYADCLHWCLPGVPDTWNELLYARIIALS---- 470
PtrXOAT6 SDPLKYADCLHWCLPGLPDTWNELLYTYIISRT---- 446
PtrXOAT3 ADPKIYADCIHWCLPGLPDTWNEFLYTHIISHT---- 418
ESK1 ADPNTYADCIHWCLPGLPDTWNEFLYTRIISRS---- 487
PtrXOAT1 ADPETYADCIHWCLPGLPDTWNEFIYTRIISRT---- 490
PtrXOAT2 ADPATYADCIHWCLPGLPDTWNEFLYTRIISRT---- 487
PtrXOAT9 ANPVSYADCVHWCLPGLQDTWNELLFAKLFYP----- 437
PtrXOAT10 ANPVSYADCVHWCLPGLQDTWNELLFAKLFYP----- 438
PtrXOAT11 SKPSSYADCIHWCLPGVPDVWNELLYAHIINL----- 409
PtrXOAT12 SNPKSYSDCIHWCLPGVPDVWNELLFHLL-------- 447
:.* .:**:***:**: *.**.:: :
Start of the DUF231 domain
DXXH motif
End of the DUF231 domain

S2 Fig
